# Interactions Between Tryptase-Positive Mast Cells and Melanin-A^+^ Cells in the Microenvironment of Cutaneous Melanoma

**DOI:** 10.3390/ijms262311313

**Published:** 2025-11-22

**Authors:** Dmitrii Atiakshin, Grigory Demyashkin, Kirill Silakov, Aleksandra Prikhodko, Vladimir Shchekin, Alexander Alekhnovich, Lyudmila Grivtsova, Demyan Davydov, Ilya Klabukov, Denis Baranovskii, Sergei Ivanov, Daniel Elieh-Ali-Komi, Igor Buchwalow, Markus Tiemann, Andrey Kostin, Petr Shegay, Andrey Kaprin

**Affiliations:** 1 Research and Educational Resource Center for Immunophenotyping, Digital Spatial Profiling and Ultrastructural Analysis Innovative Technologies, RUDN University, 6 Miklukho-Maklaya St., 117198 Moscow, Russia; path.silakov@gmail.com (K.S.); prikhodko_at@pfur.ru (A.P.); dr.shchekin@mail.ru (V.S.); alekhnovich_av@pfur.ru (A.A.); buchwalow@pathologie-hh.de (I.B.); andocrey@mail.ru (A.K.); 2 Research Institute of Experimental Biology and Medicine, Burdenko Voronezh State Medical University, 394036 Voronezh, Russia; 3 National Medical Research Radiological Center of the Ministry of Health of the Russian Federation, Koroleva St. 4, 249036 Obninsk, Russia; dr.dga@mail.ru (G.D.); grivtsova@mail.ru (L.G.); doc.baranovsky@gmail.com (D.B.); ivanov.obninsk@mail.ru (S.I.); dr.shegai@mail.ru (P.S.); kaprin@mail.ru (A.K.); 4 Laboratory of Histology and Immunohistochemistry, Institute of Translational Medicine and Biotechnology, IM Sechenov First Moscow State Medical University (Sechenov University), 119048 Moscow, Russia; 5 Columbia University, 116th and Broadway, New York, NY 10027, USA; dd3288@columbia.edu; 6 Institute of Allergology, Charité—Universitätsmedizin Berlin, Corporate Member of Freie Universität Berlin and Humboldt-Universität Zu Berlin, 10117 Berlin, Germany; daniel.elieh-ali-komi@charite.de; 7 Fraunhofer Institute for Translational Medicine and Pharmacology ITMP, Immunology and Allergology, 12803 Berlin, Germany; 8 Institute for Hematopathology, Fangdieckstr. 75a, 22547 Hamburg, Germany

**Keywords:** mast cells, cutaneous melanoma, tryptase

## Abstract

Cutaneous melanoma remains one of the most aggressive tumors, yet the role innate immunity plays in its progression remains poorly understood. Effector elements with high regulatory potential, capable of both promoting and inhibiting tumor growth—mast cells (MCs), are of particular interest. This includes quantitatively characterizing the interactions between tryptase-positive mast cells (MCs) with atypical Melanin—A^+^ cells and describing their spatial phenotype, in relation to the stage of cutaneous melanoma. A retrospective analysis was carried out on samples retrieved from 128 patients with cutaneous melanoma (AJCC 8th edition: IA–IIID). Histological analysis, histochemistry (toluidine blue, Giemsa), and diplex /multiplex IHC for tryptase and Melan-A were performed; as well as Fluorescence imaging, 3D reconstructions and quantitative mapping in QuPath v 0.6.0. Proximity was assessed by the nucleus-to-nucleus distance: <10 μm (contact), 10–20 μm (paracrine zone), >20 μm (out of interaction). The relative amount of MCs in the intratumoral zone was lower than in the intact dermis, with a simultaneous increase in their absolute density per mm^2^ in the melanoma microenvironment, maximum in the peritumoral area and most pronounced at stage II. Three types of interactions were identified: (i) juxtaposition without secretion, (ii) degranulation of MCs directed to tumor cells, (iii) melanosecretion of Melanin—A^+^ cells directed towards MCs, followed by phagocytosis of melanocores. An inverse intratumoral connection between the number of MCs and the number of Melanin—A^+^ cells was noted; MCs with elongated forms, extensive contacts and polarized tryptase secretion, including granule localization near/at the nuclei of adjacent cells, were frequently observed. The obtained data indicate stage-region-dependent bidirectional cross-talk between melanin and MCs, forming tissue spatial signals, potentially useful as biomarkers and targets for personalized therapy.

## 1. Introduction

Cutaneous melanoma remains one of the most malignant and rapidly progressing tumors, with an ever-rising incidence rate [1,2,3]. The effectiveness of immune responses is critical for the early control of neoplastic changes and the subsequent formation of robust antitumor immunity [4,5,6].

Mast cells (MCs) occupy a special place among the cells involved in the immune surveillance, being long-living effector components of the immune system with a pronounced regulatory potential due to a combination of receptor activity and secretory functions [7,8,9]. These cells take part in a wide range of physiological and pathological processes, from inflammation and tissue remodeling to carcinogenesis [10,11].

The secretory granules of MCs contain many biologically active substances, such as specific proteases, mediators and cytokines, capable of influencing cells of the tumor microenvironment and modulating local immune responses [12,13,14].

Mast cells (MCs) play a crucial role in regulating local homeostasis within specific tissue microenvironments [15,16,17].

This is facilitated by their expression of a wide range of receptors, which enable highly sensitive and selective responses to both external and internal stimuli. Furthermore, MCs possess the ability to selectively secrete diverse classes of mediators, including distinct cytokine and chemokine profiles, thereby exerting a targeted influence on the immune and stromal components of their specific microenvironment [7,8].

The effector toolkit of MCs comprises three principal classes of mediators: pre-formed mediators stored in granules, newly synthesized lipid-derived mediators, and a diverse array of cytokines, chemokines, and growth factors produced upon stimulation to modulate physiological and immune responses [18,19]. A particularly significant function of MCs is their contribution to the proinflammatory milieu. They regulate the status of numerous immune and stromal cells, as well as the extracellular matrix of connective tissues, underscoring their central role in tissue inflammation and remodeling [20,21,22,23,24,25,26,27,28,29]. At the same time, this high functional variability of MCs significantly complicates understanding their role in tumor development [30,31,32]. Despite the fact that numerous studies have been carried out on the infiltration of MCs into melanoma tissue, it remains unclear whether their accumulation leads to increased disease progression or to the inhibition of tumor growth [33,34,35].

Existing studies describe both the pro-tumor effects of MCs, associated with the secretion of growth factors, chemokines, and extracellular matrix remodeling [36,37,38], and the opposite, antitumorogenic, mediated by the release of specific proteases, primarily tryptase, and the suppression of HMGA1 protein expression, which correlates with a more favorable prognosis for patients [39,40,41].

Thus, the infiltration of mast cells into tumor growth zones is considered an important part of the immune response to melanoma. It is known that the functional activity of immune cells is closely related to their spatial distribution in tissues [42,43]. This highlights the need to study the topography of MCs at different stages of melanoma, as well as to analyze their interactions with tumor cells of melanocytic origin using spatial methods that have only been used to a limited extent to date.

The aim of this study is to spatially phenotype and map the interactions of tryptase-positive mast cells in the tumor microenvironment with atypical Melan-A^+^ cells. Analysis of morphological features of colocalization and targeted degranulation will provide a deeper understanding of the mechanisms regulating tumor growth and the potential role of mast cells in remodeling the melanoma microenvironment.

## 2. Results

### 2.1. Histological Study

**Figure 1:**
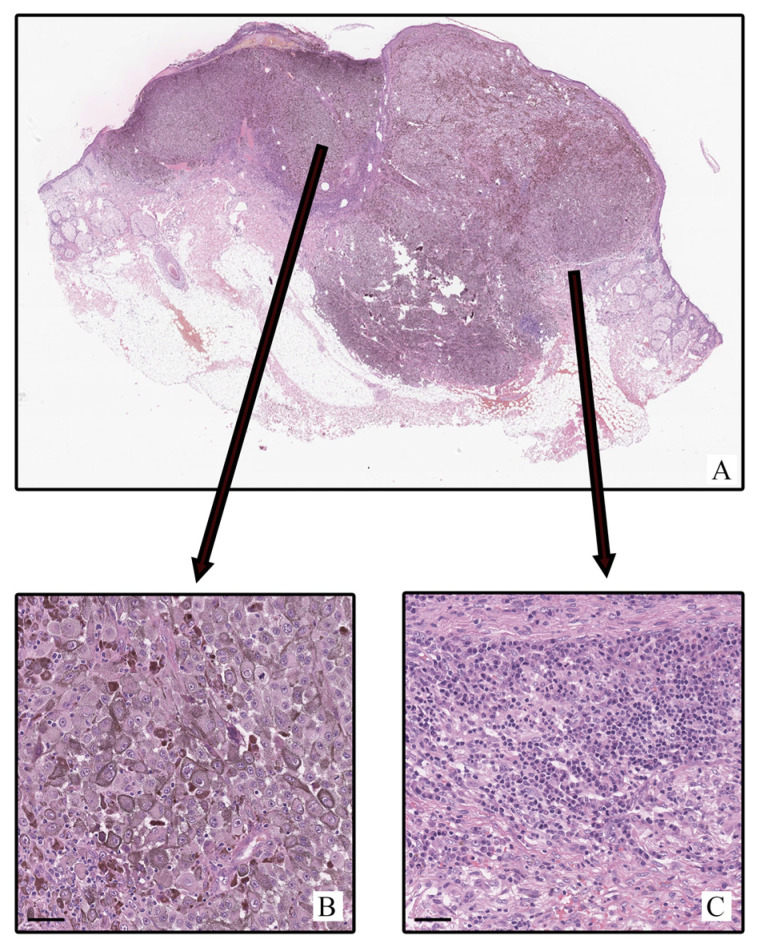
Patient R, male, 63 years. Invasive melanoma, 8721/3—nodular subtype, Breslow thickness 4.7 mm (pT4b). (**A**)—histoscan; (**B**)—tumor; (**C**)—peritumoral tissue. Staining: hematoxylin and eosin, magnification ×200. Scale: 5 μm.

Morphological features characteristic of cutaneous melanoma were found in all tumor tissue samples examined (n = 128; 100%) (Figure 1). Neoplastic cells formed irregular nests, sheets, and cords, infiltrating the dermis and, in some cases, extending into the subcutaneous fat. Tumor architecture varied from nodular to diffuse depending on the histological subtype.

Tumor cells were distinguished by pronounced cytological polymorphism: oval, polygonal, and spindle-shaped forms with moderately eosinophilic cytoplasm, often containing coarse melanin granules, were observed. Cell nuclei were enlarged, hyperchromatic, with irregular contours and prominent nucleoli. The tumor stroma had varying degrees of fibrosis and inflammatory infiltration represented by the presence of lymphocytes, plasma cells, and histiocytes. In some cases, eosinophils, neutrophils, areas with edema, and vascular congestion were observed. In some samples, areas of coagulative necrosis were determined, mainly in large and deeply invasive foci. Epidermal ulceration was observed in some cases and was accompanied by reactive hyperplasia at the periphery. Mitotic figures, including atypical ones, were observed at all stages, while mitotic activity varied depending on the degree of invasion and histological subtype. According to the AJCC classification (8th edition), the stages varied from superficially invasive forms (IA–IIB) to deeply infiltrating tumors with signs of vascular and lymphatic invasion by satellites, or intratissue metastases (IIIC–IIID).

### 2.2. Histochemical Analysis

**Figure 2:**
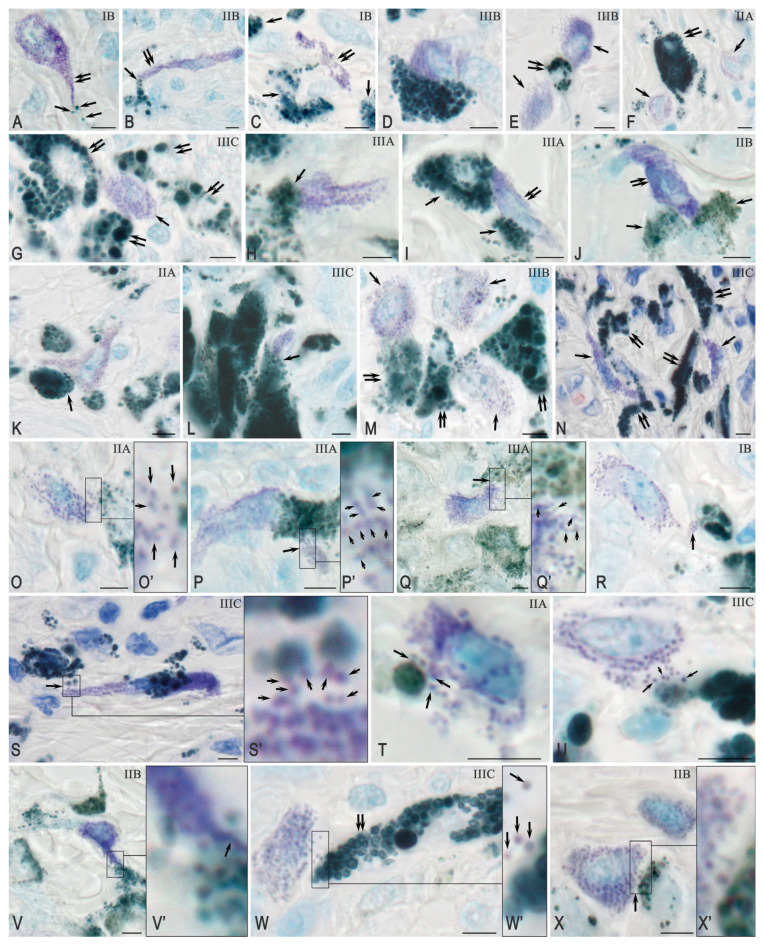
Interaction of mast cells (MCs) with atypical cutaneous melanocytes: colocalization and granule exocytosis. Method: (**N**,**S**,**T**,**V**)—Giemsa staining; others—toluidine blue. (**A**) MC projection (double arrow) towards melanocores (single arrow). (**B**) The pole of an elongated MC (double arrow) contacts an atypical melanocyte (arrow). (**C**) MC (double arrow) near a cluster of atypical melanocytes (single arrow). (**D**) Extensive contact between MCs and a melanin-containing cell. (**E**,**F**) Secretion of melanocores (double arrow) around which MCs accumulate (arrow). (**G**) Round MCs (arrow) among melanoma cells. (**H**) Contact between an MC (arrow) and a melanoma cell. (**I**,**J**) MC (double arrow) contact with two cells (arrow). (**K**,**L**) MC among atypical cells, contact with one of them (arrow). (**M**,**N**) Multiple colocalizations of MCs (arrow) with other cells (double arrow). (**O**–**Q**) Targeted secretion of MC granules towards melanoma cells (arrow). (**O’**–**Q’**) enlarged fragments of (**O**–**Q**). (**R**) Macrovesicle with granules near a cell (arrow). (**S**,**T**,**U**) Contact of MC granules with melanocores (arrow). (**S’**) enlarged fragments of (**S**). (**V**) Outgrowth of MCs with granules (arrow) towards a melanoma cell. (**V’**) enlarged fragments of (**V**). (**W**) Release of MC granules (single arrow) towards a cell (double arrow). (**W’**) enlarged fragments of (**W**). (**X**) Local secretion of MC granules (arrow). (**X’**) enlarged fragments of (**X**). The melanoma stage is indicated in the upper right corner. Scale: 5 μm.

The identification of mast cells (MCs) and determination of their distribution within the melanoma and its microenvironment, as well as within the dermis, was performed with toluidine blue staining. MCs were located in close proximity to atypical melanocytes, indicating their involvement in the formation of specific cellular interactions within the tumor microenvironment (Figure 2A–C).

Selective localization of MCs and atypical cells was noted: their spatial proximity was observed even in areas of the dermis with a low density of other cellular elements (Figure 2C–F). In some cases, clusters of MCs were observed near groups of invasive atypical melanoma cells (Figure 2E–G).

**Figure 3:**
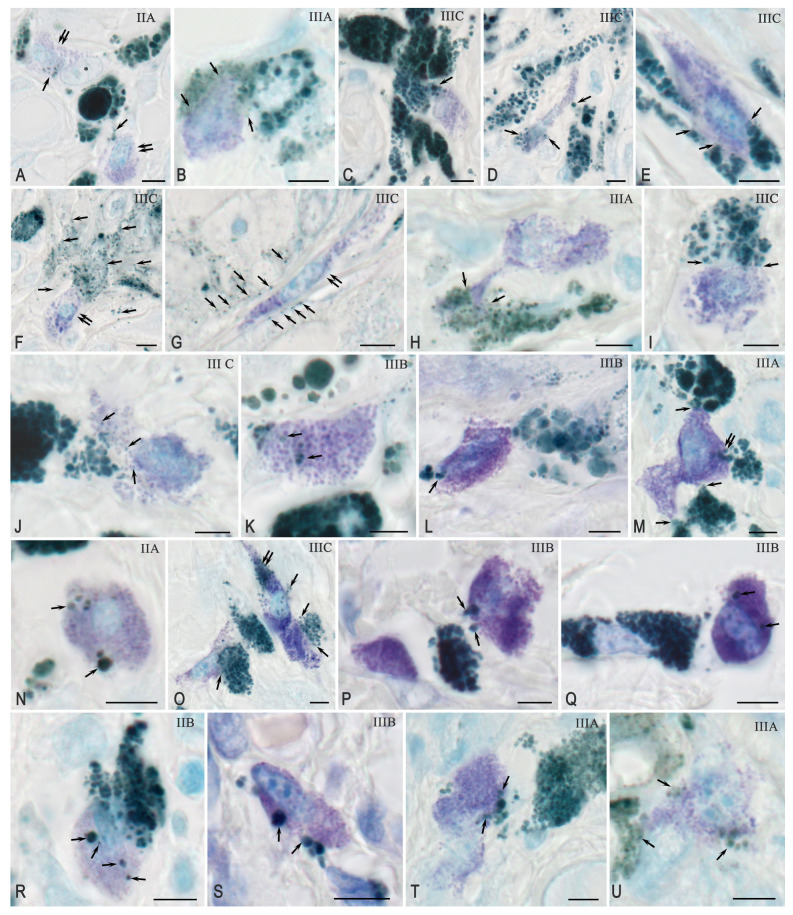
Interaction between mast cells (MCs) and atypical melanocytes: melanosecretion and phagocytosis of melanocores. Methods: (**L**,**M**,**O**–**Q**,**S**,**T**)—Giemsa staining; others—toluidine blue. (**A**–**I**) Secretion of melanocores by melanoma cells into the extracellular matrix directed towards MCs: melanocores (single arrow) directed to MCs (double arrow) (**A**); tiny melanocores affecting MCs (arrow) (**B**); cytoplasmic outgrowth of melanocyte with the transportation of large melanocores (arrow) (**C**); targeted secretion to different parts of the MC cytoplasm (arrow) (**D**); MCs surrounded by atypical melanocytes with targeted secretion (arrow) (**E**); Dissemination of dust-like melanocores (single arrow) into the MC microenvironment (double arrow) (**F**,**G**); Accumulation of tiny melanocores around a MC process (arrow) (**H**); Selective secretion of melanin into the MC locus (arrow) (**I**). (**J**–**U**) Recruitment of melanocores into MCs: small (**J**) and large (**K**) pigmented structures in the MC cytoplasm (arrow); phagocytosis of large melanocores (arrow) (**L**); multilocus interaction (single arrow) and transport into the cytoplasm (double arrow) (**M**); melanocores of different sizes in the MC cytoplasm (arrow) (**N**); intense interaction with penetration into the cytoplasm (single arrow, double arrow) (**O**); initial stages of melanocore recruitment (arrow) (**P**); Secretion of melanocores and pigment influx into MCs (arrow) (**Q**); penetration of melanocores of various sizes into the cytoplasm of MCs (arrow) (**R**); colocalization of large melanocores with MCs and transport embedded (arrow) (**S**,**T**); active action of melanocores with signs of MC apoptosis (arrow) (**U**). Scale bar: 5 µm.

Three types of interactions between MCs and atypical melanoma cells have been identified:

Close location without signs of secretory activity (Figure 2C–N);

Secretion of MC granules in the direction towards tumor cells (Figure 2O–X);

Secretion of melanin granules by atypical cells in the direction of the MCs (Figure 3A–I).

Melanin granules of varying sizes were visualized in the MC cytoplasm, corresponding to the stages of pigmentation influx from atypical cells (Figure 3J–T). In individual cases, the interaction was accompanied by morphological signs of mutual apoptosis (Figure 3U).

In most observations, when MCs were located among a group of atypical cells, preferential colocalization with one of them was observed (Figure 2K–N). Several large nucleoli were visualized in the nuclei of MCs (Figure 2M).

MCs exhibited secretory activity towards atypical cells, using various degranulation mechanisms (Figure 2O–U). MCs formed thin cytoplasmic projections that made contact with atypical melanoma cells (Figure 2V). MC secretory granules were localized in specific areas of the atypical cells’ surfaces, regardless of their size—both large (Figure 2W) and small (Figure 2X).

In most samples, the presence of melanin granules and melanocores of various sizes could be observed both around and inside the MCs (Figure 3A–I), in some places with the formation of cytoplasmic projections containing Melanosomes (Figure 3C). In individual microscopic specimens, secretion of tiny melanocores diffusing into the surrounding tissue and into the MC was detected (Figure 3F–I). At all stages of melanoma, melanin-containing structures of varying sizes were detected in the cytoplasm of the MCs, which points to phagocytosis (Figure 3J–T).

In a number of patients, primarily with stage III melanoma, elevated eosinophil counts were detected in the tumor microenvironment (Supplementary S1A–M). Eosinophils were visualized using toluidine blue staining and made contact with both MCs (Supplementary S1A–D) and atypical melanocytes (Supplementary S1E–I). Triple cell associations were observed, including MCs, eosinophils, and tumor cells (Supplementary S1J–M).

### 2.3. Immunohistochemical Analysis

#### 2.3.1. Quantitative and Cytological Features of Tryptase-Positive Mast Cells Associated with Cutaneous Melanoma

**Figure 4:**
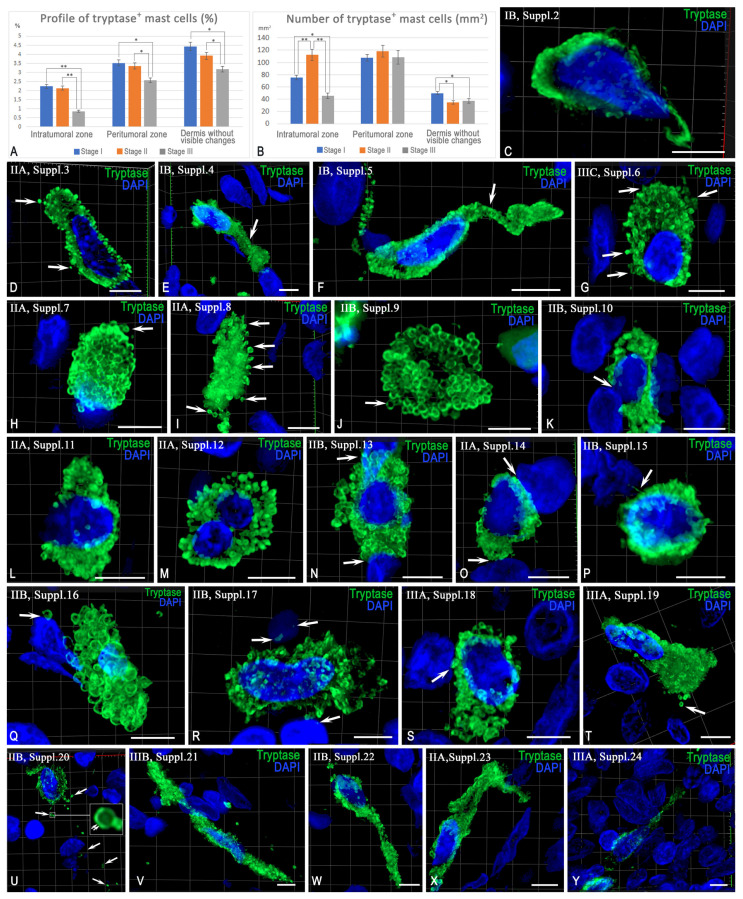
Phenotypes of tryptase-positive mast cells (MCs) in the tumor microenvironment. Method: (**C**–**Y**)—immunohistochemical detection of tryptase, 3D reconstruction using Z-stacks (see Supplementary S2–S24). (**A**,**B**) MC content in the intratumoral zone (ITZ), peritumoral zone (PTZ) and intact dermis (ID) in relative (%) and absolute (per mm^2^) units. (**C**,**D**) Uneven (**C**) and uniform (**D**) filling of the cytoplasm with granules and their transport into the extracellular matrix (arrow). (**E**,**F**) Elongated MCs with cytoplasmic processes (arrow). (**G**,**H**) Peripheral location of the nucleus and secretory activity (arrow). (**I**,**J**) Tryptase-positive MC scaffolds without nuclei filled with large granules (arrow). (**K**) Colocalization of the MC nucleus with the nucleus of the neighboring cell (arrow), zone without granules. (**L**,**M**) Binuclear MC forms. (**N**,**O**) Colocalization of granules with the nuclei of neighboring cells (arrow). (**P**–**S**) The entry of tryptase into the nuclei of neighboring cells (arrow), including transport in granules (**Q**,**R**). (**T**) Peripheral MC nucleus and exit of granules from the opposite pole (arrow). (**U**) Active secretion of tryptase into the extracellular matrix (arrow); granules maintain peripheral localization of tryptase (double arrow). (**V**–**Y**) Elongated MC forms provide contacts with other cells at a distance. The melanoma stage and the corresponding Supplementary number are indicated in the upper left corner; * and **—*p* < 0.05 and 0.01, respectively.

Defining the MC content profile relative to other skin cells revealed that the highest level was observed in the dermis without signs of pathological changes, where MCs constituted approximately 5% of the total cell count (Figure 4A). In the melanoma microenvironment, regardless of the disease stage, the relative MC profile was significantly lower compared to the intact dermis, which is best expressed in the intratumoral region (Figure 4A). This is associated with an increase in overall cellular density stemming from the proliferation of atypical cells, stromal elements, and the migration of immunocompetent cells. Thus, while the overall MC count increased, their relative content decreased.

Calculating the number of MCs per unit area confirmed an increase in their density (per mm^2^) compared to intact dermis, which was most pronounced in stage II melanoma (Figure 4B). Maximum density values were recorded in the peritumoral region (Figure 4B).

The morphological phenotypes of tryptase-positive MCs in the tumor microenvironment are shown in Figure 3. Secretory granules were either uniformly distributed in the cytoplasm or localized in separate areas. Whole tryptase-positive granules of varying sizes were frequently observed to be released into the extracellular matrix (Figure 4C,D; Supplementary S2 and S3). In the tumor microenvironment, MCs often acquired an elongated shape due to the formation of cytoplasmic outgrowths, which is particularly clearly demonstrated in the 3D models (Figure 4E,F; Supplementary S4 and S5).

Compared with the intact dermis, the nuclei of MCs were more often located peripherally. Evidence of denucleation was observed, including the formation of large anucleate cytoplasmic fragments that retained the ability to secrete tryptase (Figure 4G–J; Supplementary S6–S9). Cases of close proximity of the MC nucleus to the nucleus of the adjacent cell were observed; in the contact zone, a section of the cytoplasm was freed from secretory granules, which ensured the maximum proximity of the nuclei (Figure 4K; Supplementary S10). Binucleated cells were also detected in the tumor-associated MC population (Figure 4L,M; Supplementary S11 and S12). Tryptase-positive granules were often localized near the nuclei of adjacent cells, forming areas of dense colocalization (Figure 4N–P; Supplementary S13–S15). In some observations, granules were detected within the nuclei of adjacent cells (Figure 4Q–S; Supplementary S16–S18). Some MCs showed signs of cytological polarization with the nucleus localized at one pole of the cell and granule accumulation in the zone of active degranulation (Figure 4T; Supplementary S19). Secretory granules that left the cytoplasm were located at a significant distance from the cell, maintaining the peripheral distribution of tryptase (Figure 4U; Supplementary S20).

Some MCs took on a highly elongated shape, extending over significant distances in the tumor microenvironment and contacting a large number of cells and extracellular structures (Figure 4V–Y; Supplementary S21–S24).

#### 2.3.2. Interaction Between Tryptase-Positive Mast Cells and Atypical Melan-A^+^ Melanoma Cells

**Figure 5:**
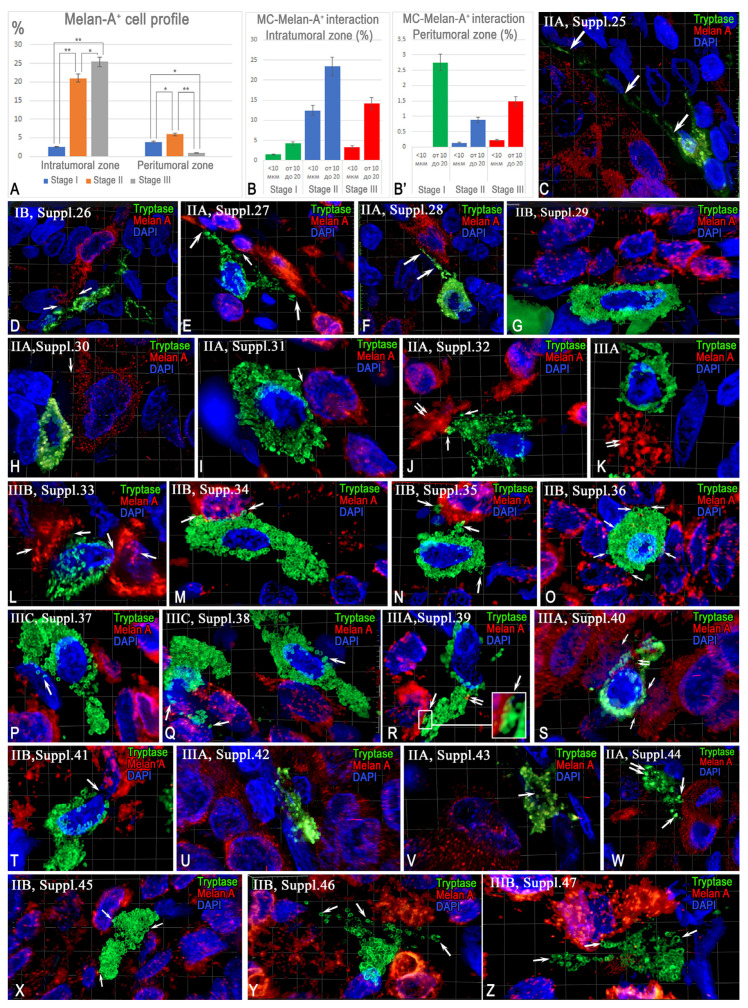
Interaction of mast cells (MC) with atypical Melan-A^+^ melanoma cells. Method: (**C**–**Z**)—diplex Immunohistochemical staining of tryptase and Melan-A, nuclei—DAPI; 3D reconstruction from Z-stacks (Supplementary S25–S47). (**A**) Profile of Melan-A^+^ cells in the intratumoral and peritumoral zones. (**B**,**B’**) Frequency of juxtacrine and paracrine colocalization of MCs with Melan-A^+^ cells at different stages of melanoma. (**C**–**G**) Demarcating localization of MCs: elongated shapes (arrow) and contacts with atypical cells, including extensive contact zones (arrow). (**H**–**K**) MCs with predominant tryptase secretion to one atypical cell (arrow), signs of Melan-A^+^ cell apoptosis (double arrow). (**L**–**Z**) Multiple interactions of MCs with multiple cells: contacts (arrow), tryptase secretion to the nuclei of adjacent cells (arrow), penetration of tryptase into granule content (arrow, double arrow), and phagocytosis of Melan-A^+^ structures (double arrow). MCs demonstrate encirclement by atypical cells and accumulation of melanocores (arrow, double arrow). Variants of MC apoptosis include loss of nucleus (arrow), separation of tryptase-positive cytoplasmic fragments (double arrow), and autonomous secretion of tryptase to Melan-A^+^ cells (arrow). Melanoma stage and Supplementary number are indicated in the upper left corner; * and **—*p* < 0.05 and 0.01, respectively.

The profile of Melan-A^+^ cells increased with disease progression from stage I to stage III in the intratumoral zone. (Figure 5A). In the peritumoral zone, the highest density of atypical melanocytes was observed at stage II melanoma (Figure 5A). The intensity of juxtacrine and paracrine colocalization of MCs with atypical melanoma cells was at its highest at stage II of the disease: approximately 40% of MCs in the intratumoral zone interacted with Melan-A^+^ cells (Figure 4B). At stage III, the frequency of such interactions decreased compared to stage II, but remained higher than at stage I (Figure 5B). In the peritumoral zone, the highest activity level of paracrine interactions between MCs and Melan-A^+^ cells was observed at stage I and exceeded the values recorded at stages II and III (Figure 5B’). Histo-topographic analysis revealed a frequent location of MCs at the border in relation to atypical melanocytes (Figure 5C–G). In the areas of colocalization, MCs took on an elongated shape, surrounding atypical cells (Figure 5C; Supplementary S25), or were located parallel to them, forming extended areas of contact (Figure 5D,E; Supplementary S26 and S27). Elongated cytoplasmic processes of MCs were noted, adjacent to the surface of atypical cells (Figure 5F; Supplementary S28). Oval-shaped MCs were often localized at the border of clusters of atypical cells (Figure 5G; Supplementary S29). MCs secreted tryptase in granules. The secretion was directed and could be oriented toward one of several adjacent atypical cells (Figure 5H–K). In some observations, morphological signs of apoptosis were observed in atypical cells (Figure 5J,K). Three-dimensional models detail the spatial aspects of the colocalization of MCs and atypical cells (Supplementary S30–S32). In addition to monotargeted tryptase secretion, cases of MC degranulation towards two or more atypical cells were observed (Figure 5L–Z). Some atypical cells showed signs of apoptosis upon interaction with MCs (Figure 5L; Supplementary S33).

The colocalization zones of MCs with atypical cells were characterized by an extensive contact area (Figure 5M; Supplementary S34) and targeted tryptase secretion (Figure 4N; Supplementary S35). In some cases, MCs were completely surrounded by tumor cells (Figure 5O; Supplementary S36). In a number of observations, signs of tryptase entry into the nuclei of adjacent cells were determined, including the data obtained from the three-dimensional reconstruction (Figure 5P,Q; Supplementary S37 and S38). Tryptase secretion could be directed to specific areas of the atypical cell surface (Figure 5R; Supplementary S39). The interaction of atypical melanoma cells with MCs was accompanied by the secretion of small melanocores, which were subsequently phagocytosed (Figure 5S; Supplementary S40). In some other cases, contacts between MCs and atypical cells with closely spaced nuclei were recorded (Figure 5T; Supplementary S41). Sometimes, the interaction was accompanied by morphological signs of MC apoptosis while maintaining targeted tryptase secretion towards the atypical cells (Figure 5U–Z; Supplementary S42–S47).

## 3. Discussion

The characteristic patterns of spatial distribution of mast cells and their contacts with atypical cells indicate their crucial role in the formation of the tumor microenvironment, as well as their involvement in the carcinogenesis of skin melanoma at all stages of tumor growth.

Resident skin MCs are known to carry out constant immune surveillance, responding to the production and migration of antigens and changes in local tissue homeostasis [44]. Due to this function, MCs are among the first cell populations to respond to the appearance of tumor elements and modify their own phenotype during the formation of neoplastic transformation [36]. A number of studies emphasize that MCs can trigger and modulate antitumor immune responses [45].

It has previously been shown that MCs closely colocalize with atypical melanoma cells and specifically secrete specific proteases, regardless of the stage, extent, and intensity of the tumor process [12]. In this present study, we mapped the spatial phenotype of MCs in stages I–III melanoma, including an assessment of their content in various zones of the microenvironment and an analysis of the histo-topographic characteristics of intercellular interactions. It was found that the distribution of MCs and Melan-A^+^ cells, as well as the intensity of their contacts, varied significantly between patients, even within the same clinical stage. These differences reflect the individual organization of the immune landscape and the target activity of MCs. Particularly noteworthy is the inverse relationship between the number of MCs and the number of Melan-A^+^ cells within the tumor itself: an increase in one population was accompanied by a decrease in the other.

This ratio is consistent with data on melanin-mediated suppression of MC functions by atypical melanocytes [46]. It has been shown that melanin leads to a decrease in the proliferation of mast cells, induces apoptosis, and reduces their degranulation potential and the production of proinflammatory cytokines, forming a local immunosuppressive environment [46]. Practically the same results were obtained by [36], where the absence of factors inducing MC degranulation in melanoma cells was noted. In contrast, pharmacological stimulation of MC degranulation is associated with enhanced antitumor response, decreased tumor growth, and improved survival [14,47].

We observed the presence of melanosomes in the cytoplasm of mast cells, confirming the possibility of its direct influence on the functions of these cells, which is particularly interesting in the context of neoplasia. Melanin is capable of penetrating the nucleus and inducing tryptase translocation through the nucleolemma, which is accompanied by modification of histone H3 and disruption of the lamin B1 structure, as well as potential epigenetic effects [46]. These facts support the hypothesis that intense melanogenesis and melanosecretion can functionally “switch off” the innate immune system through MC inactivation; therefore, melanin destruction is theoretically capable of restoring the antitumor potential of MC. Additionally, a decrease in the expression of tryptase (TPSAB1) and chymase (CMA1) genes has been reported in MC compared to normal skin and basal cell carcinoma [36].

We simultaneously documented evidence of active MC effects on atypical cells in both paracrine and juxtacrine formats. Three-dimensional patterns clearly revealed extensive contact zones, where MCs’ cytostatic effects may be mediated. Similar ultrastructural features have been described in cervical paragangliomas, where MCs form thin cytoplasmic protrusions for stable intercellular positioning [48]. Contact-dependent inhibition of melanoma cells by MCs has also been demonstrated in studies on HMGA1 biogenesis [41].

Tryptase plays a key role among the secretory factors of MCs [35,49]. Endocytosis of tryptase by atypical melanocytes is followed by its nuclear localization, H3 modification, and destruction of lamin B1. Additionally, its effects on mRNA stability through interaction with non-coding RNAs and suppression of EGR135 oncogene expression with subsequent morphological changes in tumor cells have been described [49]. Higher levels of serum tryptase and its mRNA are associated with a less aggressive course of the disease [40]. The antitumor effects are likely complemented by the activity of other MC proteases, including chymase, which reduces the growth and proliferation of tumor cells [50,51], as well as the action of histamine, which limits melanoma growth and accelerates tumor aging [17,52].

Proinflammatory aspects should also be considered; increased PAR-2 expression in tumor microenvironment components may determine the effects of tryptase, alongside persistent inflammation and increased IL-8 production [53,54]. In addition to specific proteases, MCs are capable of targeted secretion of nonspecific enzymes (e.g., granzyme B) towards atypical cells. Our material demonstrates evidence of targeted tryptase secretion to target cells in the microenvironment.

The distribution of immunocompetent cells across tumor zones documented in our study is partly consistent with existing scientific literature data: intratumoral MCs are generally fewer in number, while peritumoral infiltrate is more pronounced [1]. Moreover, even in the presence of contact interactions between MCs and atypical cells within the tumor node, melanoma progression may persist [50].

Taken together, our results indicate that MCs exert a significant effect on Melan-A^+^ cells through juxtacrine and paracrine mechanisms already at the early stages of neoplastic lesion formation, as well as during tumor invasion into the dermis. The antitumor activity of MCs is also confirmed in a number of studies. vivo studies [34,39,55]. The overall effect of MCs on tumor growth and progression is determined not only by the direct effect on melanocytic cells, but also by indirect regulation of other components of the microenvironment [50]. Finally, the recorded denucleation of MC may indicate the formation of extracellular traps (extracellular traps), which is consistent with previously described mechanisms of antitumor activity [14].

## 4. Materials and Methods

This retrospective, multicenter, cohort study was observational and analytical in design and was conducted spanning 2023–2024 years at the National Medical Research Radiological Center (Moscow, Russia). Potential patient cases were initially identified through a search of the electronic medical record systems at both institutions using International Classification of Diseases, 10th Revision (ICD-10) codes corresponding to the primary diagnosis C43—Malignant melanoma of skin.

The study included data from 128 patients with a confirmed diagnosis of melanoma. Medical records, primarily case histories, were used for analysis (Table 1). For all patients, anamnesis, clinical manifestations, disease stage according to the American Joint Committee on Cancer (AJCC 8th edition) classification, the nature of surgical and conservative treatments, as well as disease outcomes, were taken into account. Surgical specimens (paraffin blocks) stained with hematoxylin and eosin were also examined, and histological subtypes of melanoma were studied.

Based on the collected data, patient groups were formed corresponding to stages IA, IB, IIA, IIB, IIC, IIIA, IIIB, IIIC and IIID according to the AJCC 8th edition classification.

Inclusion criteria: Cutaneous melanomas of the specified stages without distant metastases (M0); histological subtypes: superficial spreading melanoma (ICD-O: 8743/3; Superficial spreading melanoma), nodular melanoma (ICD-O: 8721/3; Nodular melanoma), desmoplastic melanoma (ICD-O: 8745/3; Desmoplastic melanoma); functional status according to ECOG scale 0–1.

### 4.1. Tissue Probe Staining

The tissue probes that were fixed in buffered 4% formaldehyde and routinely embedded in paraffin and subjected to standard sample preparation procedures: in accordance with current standard operating procedures, histological sections 5 μm thick were prepared for histochemical staining and histological sections 2.5 μm thick were prepared to implement monoplex and multiplex immunohistochemical staining protocols [56].

### 4.2. Immunohistochemistry (IHC) and Histochemistry

Immunohistochemistry was performed according to standard protocols. Skin melanoma tissue samples were fixed in 4% buffered formaldehyde, embedded in paraffin, and sectioned at 2.5 μm for immunohistochemical and immunofluorescent staining. Primary antibodies used included anti-tryptase (mouse monoclonal, Abcam, Cambridge, UK; ab2378, dilution 1:4000) and anti-Melan-A (mouse monoclonal, Abcam, Cambridge, UK; ab210546, dilution 1:100). For fluorescent multiplex staining, tryptase was additionally detected using a directly conjugated anti-tryptase antibody (Alexa Fluor 488, Abcam, Cambridge, UK; ab2378-AF488, dilution 1:300). For secondary antibody detection, Alexa Fluor–conjugated goat anti-mouse IgG and goat anti-rabbit IgG antibodies (Abcam, Cambridge, UK; Alexa Fluor 555 and 647, dilution 1:300) were applied. Nuclei were counterstained with DAPI (Sigma, Hamburg, Germany; 5 µg/mL), and sections were mounted with VECTASHIELD^®^ Mounting Medium (Vector Laboratories, Burlingame, CA, USA).

Visualization of HRP-linked antibodies was performed using the AmpliStain ™ 1-Step HRP Detection System (SDT GmbH, Baesweiler, Germany) with DAB (Vector Laboratories, Burlingame, CA, USA) as chromogen substrate. Histochemical stains—toluidine blue, Giemsa, and Mayer’s hematoxylin—were used according to manufacturer protocols.

Positive control—skin sections with abundant mast cells were used to verify tryptase staining; melanoma tissue known to express Melan-A served as a positive control for Melan-A immunostaining. Negative control—serial tumor sections were incubated without primary antibody or with isotype-matched control IgG (Dianova, Hamburg, Germany) at the same concentration; no staining was observed in these controls.

### 4.3. Image Acquisition

For the study of microsections stained according to histochemical and immunohistochemical protocols, light microscopy and multiplex visualization were performed using a ZEISS AxioImager.Z2 microscope equipped with Zeiss alpha Plan-Apochromat objective 100×/1.46 OilDICM27, Zeiss Objective Plan-Apochromat 150×/1.35 GlycDICCorrM27, and ZEISS Axiocam 712 color digital microscope camera and ZEISS Axiocam 712 mono digital microscope camera (Carl Zeiss Vision, Jena, Germany). In some cases, photomicrographs were obtained with a Nikon D-Eclipse C1 Si confocal microscope based on the Nikon Eclipse 90i platform (Nikon Instruments Inc., Tokyo, Japan).

### 4.4. Quantitative Analysis

Quantitative analysis was performed on whole tumor sections. Planimetric analysis was used to determine the number of search cells (MCs, immune cells, stromal cells, and atypical tumor cells) per unit area of the sections, the absolute and relative number of MCs and other cells, and the profile of mast cell-specific proteases using the QuPath v0.6.0 software product [57]. Mapping and imaging of stained sections were performed after scanning the entire histological section using a digital pathology slide scanner (fluorescence) KF-FL-005 (Ningbo, China), ×40 objective of a ScanScope CS (Leica Biosystems, Deer Park, IL, USA), and the Mantra 2 Quantitative Pathology Imaging System (Akoya Biosciences, Marlborough, MA, USA) based on an Olympus BX43 microscope. Nuclei were identified using the Stardist extension [58]. The intensity threshold for the DAPI channel was defined with a pixel classifier built into Qupath, trained on expert annotations. Further classification of segmented detections by phenotype was achieved by training a neural network with point annotations and subsequent iterative verification by a specialist. Autofluorescence was minimized using a dedicated reagent kit and by optimizing exposure time during scanning. Cell co-localization was calculated by measuring the distance between nuclear centers, taking into account the length of their semi-axes. The minimum distance, defined as the sum of the major semi-axes of the nuclei, averaged ≤10 μm. Distance categories were defined as follows: <10 μm, contacting cells; 10–20 μm, cells within the paracrine interaction zone; >20 μm, non-interacting cells. At each iteration, results were reviewed by a specialist and parameters adjusted as necessary. Quantitative data were exported from QuPath v0.6.0 to R for further visualization and analysis [59].

### 4.5. Statistical Analysis

Statistical analysis was performed using the SPSS software package (Version 13.0, IBM, Armonk, New York, USA). The results are presented as the mean (M) ± m (standard error of the mean). To assess the significance of the differences between the two groups, Student’s *t*-test or the Mann–Whitney U test in the case of a nonparametric distribution was used. Significance was considered as *—*p* < 0.05, **—*p* < 0.01.

## 5. Conclusions

The spatial phenotype of interactions between tryptase-positive mast cells (MCs) and atypical Melan-A^+^ cells in the melanoma microenvironment has significant diagnostic and prognostic potential, which can be used to individualize therapy. Histo-topographic analysis and assessment of MC tumor cell–cell contacts allow us to develop criteria for their antitumor activity and consider these parameters as promising spatial tissue biomarkers.

The identified patterns of distribution of tumor-associated MCs depending on the content of Melan-A^+^ cells confirm the participation of melanin in modulating the activity of innate immunity and indicate its possible role as a pharmacological target aimed at restoring the antitumorogenic potentials of MCs.

The use of spatially structured multiplexed detection of cellular phenotypes allowed us to clarify the morphological characteristics, topology, and functional activity of mast cells in the context of the melanoma tumor microenvironment. The obtained results contribute to a deeper understanding of the mechanisms of interaction between mast cells and melanocytes and pave the way for the development of new prognostic and translational biomarkers applicable to the assessment of therapy effectiveness and personalized treatment strategies for melanoma.

## Figures and Tables

**Table 1:** Clinicopathological Characteristics of the Patients.

Characteristic	Rate
Age	29–88 years
Average age	64.2 ± 6.8 years
Gender	
Male	57
Female	73
Histologic type	
Superficial spreading melanoma	49
Nodular melanoma	69
Desmoplastic melanoma	12
Breslow thickness	
<0.8–1.0 mm	33
>1.0–2.0 mm	29
>2.0–4.0 mm	36
>4.0 mm	32
Clark level of invasion	
I	17
II	26
III	24
IV	49
V	12
Localization of primary melanoma	
Head and neck	19
Trunk	32
Upper extremities	29
Lower extremities	48
pT	
pT1a	24
pT1b	9
pT2a	21
pT2b	9
pT3a	21
pT3b	14
pT4a	17
pT4b	15
AJCC stage	
IA	23
IB	10
IIA	12
IIB	14
IIC	14
IIIA	17
IIIB	17
IIIC	17
IIID	6

Exclusion criteria: Spitz-type lesions; prior systemic or local antitumor therapy; presence of distant metastases or recurrent disease; synchronous or metachronous multiple primary cancers; infectious diseases; autoimmune disorders.

## Data Availability

The original contributions presented in this study are included in the article/Supplementary Material. Further inquiries can be directed to the corresponding author.

## Supplementary Materials

The following supporting information can be downloaded at https://www.mdpi.com/article/10.3390/ijms262311313/s1.
