# Supplementary material for: Interactions Between Tryptase-Positive Mast Cells and Melanin-A+ Cells in the Microenvironment of Cutaneous Melanoma
Source: Int J Mol Sci. 2025 Nov 22;26(23):11313. doi: 10.3390/ijms262311313 (PMC12692133; doi:10.3390/ijms262311313)
Supplement: Supplementary file 1 [file ijms-26-11313-s001.zip › ijms-3991757-Supplementary materials+.pdf]

APPENDIX

Supplementary S1

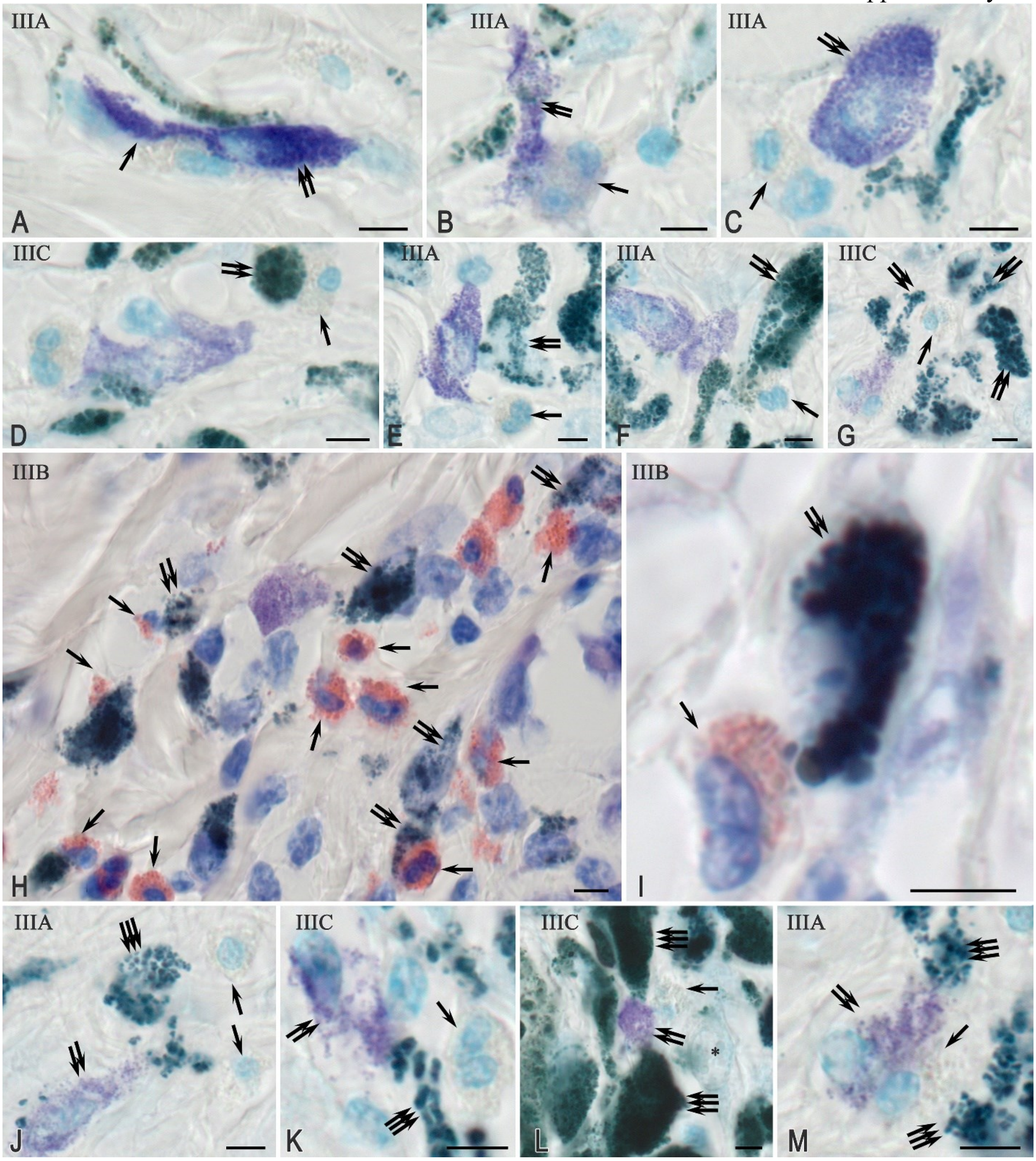


Supplementary S1. Interaction between mast cells and eosinophilic granulocytes in the tumor microenvironment of a melanoma. Method: histochemical staining with toluidine blue (A-G, J-M) and Giemsa staining (H-I). Comments. (A-D) Variants of eosinophils (arrow) colocalization with mast cells (double arrow). (D-I) Contact of eosinophils (arrow) with atypical melanocytes (double arrow). (J-M) Simultaneous colocalization of eosinophils (arrow), MCs (double arrow) and atypical melanocytes (triple arrow). In the picture (L) the tumor-associated fibroblast ( asterisk * ) is clearly visible.

| No. Supplementary | Description | Freeze frame of 3D models | Link to 3D model |
|---|---|---|---|
| S2 | Uneven filling of the mast cell cytoplasm with secretory granules showing signs of whole-granular transports into the extracellular matrix | 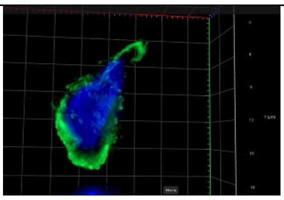 | https://disk.yandex.ru/i/ZUDG1ZkaGnxn3w |
| S3 | Uniform accumulation of secretory granules in the mast cell with signs of secretion into the extracellular matrix | 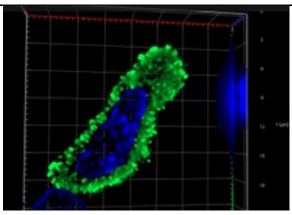 | https://disk.yandex.ru/i/3vqilLTzWTG3Ww |
| S4 | Elongated-shaped mast cells with pronounced cytoplasmic outgrowths | 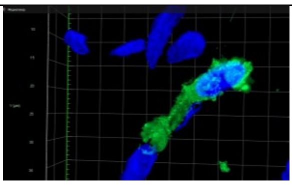 | https://disk.yandex.ru/i/-6ZKMyQD0EAkdQ |
| S5 | | 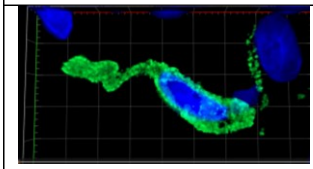 | https://disk.yandex.ru/i/0ocdDn9BcxxnGA |
| S6 | Nucleus peripheral position in the cytoplasm of mast cells | 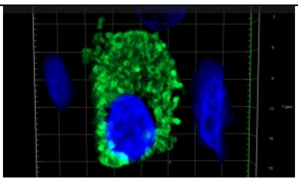 | https://disk.yandex.ru/i/i9GiIKBDTrolLg |
| S7 | | 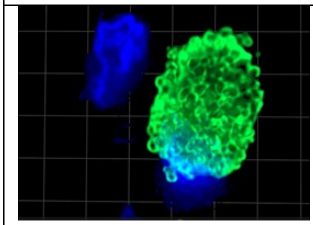 | https://disk.yandex.ru/i/D5l3E9YBOc0ruA |
| S8 | Cytoplasmic tryptase-positive scaffolds of mast cells that have lost their nuclei, filled with large | 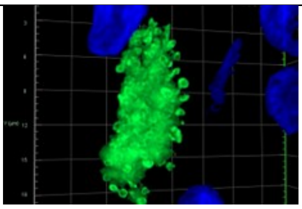 | https://disk.yandex.ru/i/dDQgfgZnNpTWFQ |

| | | | |
|---|---|---|---|
| S9 | granules, showing signs of secretory activity | 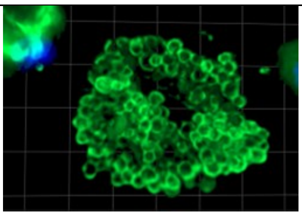 | https://disk.yandex.ru/i/jfSWfbJdUEv5GA |
| S10 | Close colocalization of the mast cell nucleus with the nucleus of a nearby cell (arrow), absence of secretory granules in the area of contact | 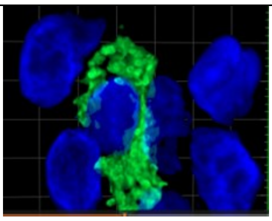 | https://disk.yandex.ru/i/Zrfw4BgiVd9G4Q |
| S11 | Binucleated mast cell variants | 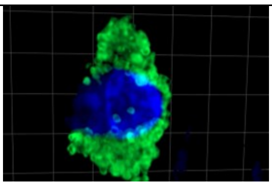 | https://disk.yandex.ru/i/wWgrrXUbd1fF0Q |
| S12 | | 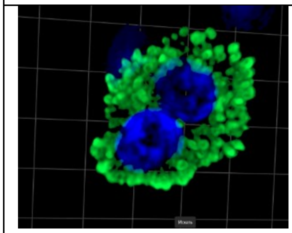 | https://disk.yandex.ru/d/2k-sUlD4cAwCiQ |
| S13 | Variants of close colocalization of mast cells' tryptase-positive granules with the nuclei of neighboring cells | 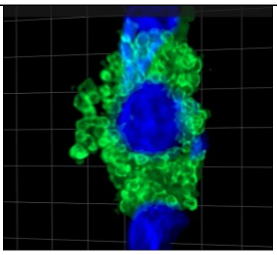 | https://disk.yandex.ru/i/R9l2WxdYdsJ9Qg |
| S14 | | 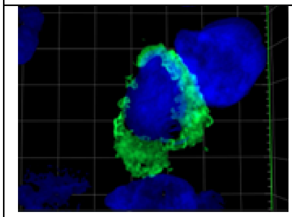 | https://disk.yandex.ru/i/64pqGg7yiHQV2g |
| S15 | Morphological equivalents of the entry of mast cell tryptase into the nuclei of neighboring cells | 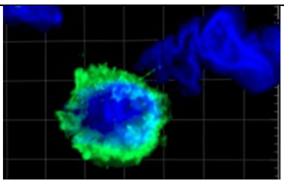 | https://disk.yandex.ru/i/-o9J6EEsUz7spg |

| | | | |
|---|---|---|---|
| S16 | | 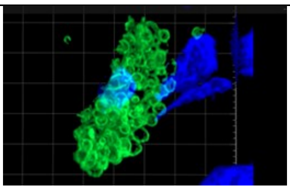 | https://disk.yandex.ru/i/u9PvWPgoTDzK5A |
| S17 | | 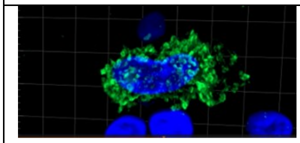 | https://disk.yandex.ru/i/qvp2oQAKe7d2Ug |
| S18 | | 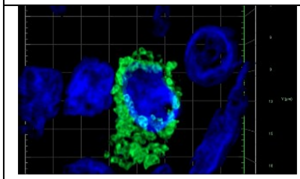 | https://disk.yandex.ru/i/JScnt-gOuXOdUw |
| S19 | Nucleus peripheral location in the cytoplasm of a mast cell with the predominant release of secretory granules at the opposite pole | 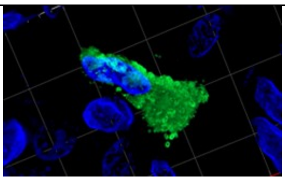 | https://disk.yandex.ru/d/GWXk0hum--PDFA |
| S20 | Active secretion of tryptase contained in granules, which travel significant distances to reach tissue microenvironment targets, into the extracellular matrix. After secretion, secretory granules maintain the peripheral intragranular localization of tryptase. | 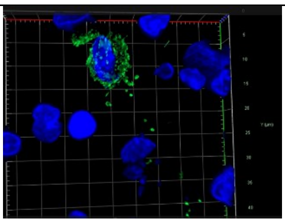 | https://disk.yandex.ru/i/U8TpZlKJ1ulvrA |
| S21 | Mast cells, possessing a pronounced elongated shape, which makes contacts with | 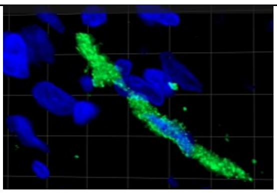 | https://disk.yandex.ru/i/CngtYqomfpTbrw |

| | | | |
|---|---|---|---|
| S22 | other cells possible over a considerable distance | 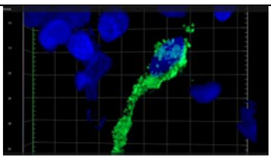 | https://disk.yandex.ru/i/10zf8zdGhWd8_g |
| S23 | | 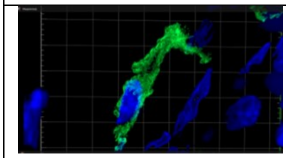 | https://disk.yandex.ru/i/NiSjxfcHH25h0Q |
| S24 | | 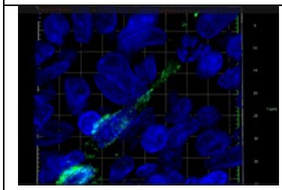 | https://disk.yandex.ru/i/85FCw2Ey-q78lA |
| S25 | Elongated mast cells (arrow) surround a cluster of atypical cells | 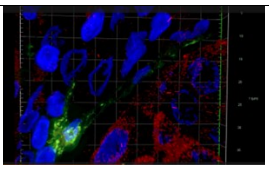 | https://disk.yandex.ru/i/yu9wmFUzN2JDuQ |
| S26 | Mast cells follow the position pattern of atypical cells and make contact with them | 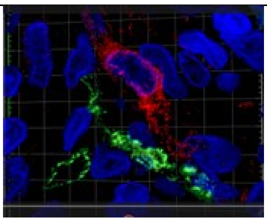 | https://disk.yandex.ru/i/8aGFUO9rsK7rIA |
| S27 | | 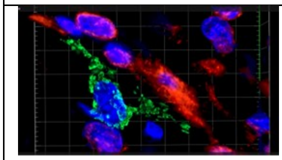 | https://disk.yandex.ru/i/dJqgxULEDSvonQ |
| S28 | The mast cell process is colocalized over a large area with an atypical melanoma cell | 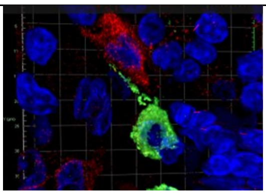 | https://disk.yandex.ru/i/d6uxX4fDiFOcJw |
| S29 | A large mast cell, filled with secretory granules, located at the border of a group of atypical cells | 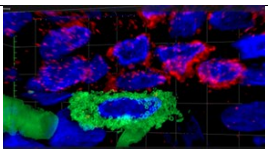 | https://disk.yandex.ru/i/jU_ZSXTEq_oalQ |
| S30 | Mast cells with predominant tryptase secretion (arrow) towards a single atypical cell. | 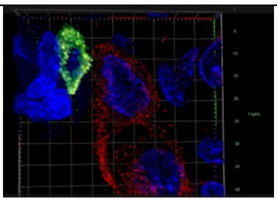 | https://disk.yandex.ru/i/kns3rlaFiOZrWw |

| | | | |
|---|---|---|---|
| S31 | | 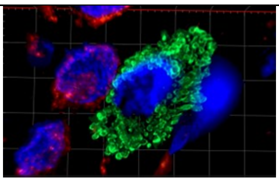 | https://disk.yandex.ru/i/4PqT5IligNWjyg |
| S32 | | 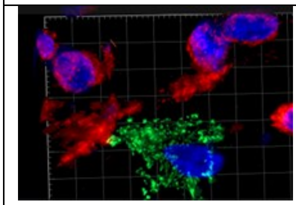 | https://disk.yandex.ru/i/Luulu80_Fve7vQ |
| S33 | Active interaction with two atypical cells (arrow), one of which has morphological signs of apoptotic changes | 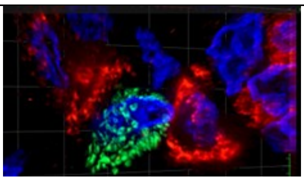 | https://disk.yandex.ru/i/roMIzBBdCrCopg |
| S34 | A large mast cell filled with a great number of large secretory granules interacts with two atypical cells, forming a broad contact area with one of them. | 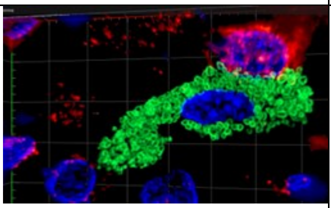 | https://disk.yandex.ru/i/RMgjN68cY3P6NQ |
| S35 | Colocalization of a mast cell with several atypical cells with the implementation of targeted secretion of tryptase towards two out of them | 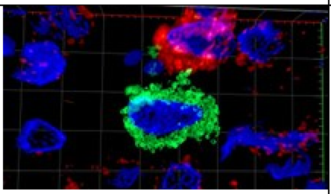 | https://disk.yandex.ru/i/r67Kv7gEy6ZtzA |
| S36 | A mast cell completely surrounded by atypical cells. Tryptase secretion into the nuclei of adjacent cells is visualized. | 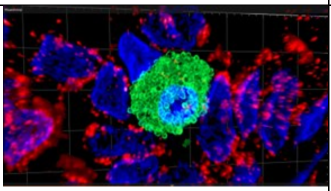 | https://disk.yandex.ru/i/LN54YmuUezCu5Q |
| S37 | Signs of mast cell tryptase entering the nuclei of | 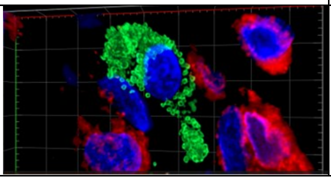 | https://disk.yandex.ru/i/Qq9Tn07F1eGYvQ |

| | | | |
|---|---|---|---|
| | neighboring cells | | |
| S38 | as part of the granules | 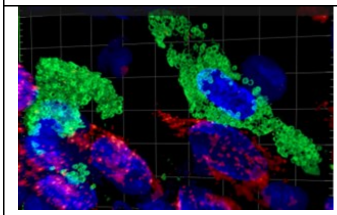 | https://disk.yandex.ru/i/BUtCEMnsJ5mSEg |
| S39 | Targeted secretion of tryptase to a specific locus of an atypical cell, as well as the initial stages of phagocytosis of Melan-A-positive structures into the mast cell, are visualized. | 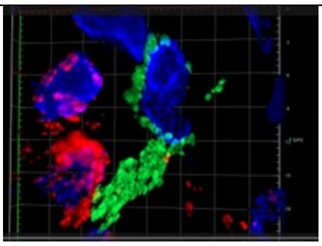 | https://disk.yandex.ru/i/l1RBK_eiSOvkvA |
| S40 | A mast cell surrounded by atypical melanoma cells. Active secretion of small Melan-A-positive melanocores by the atypical cells to the surface of the mast cell is observed (arrow), with some Melan-A-positive substance visualized in the cytoplasm. | 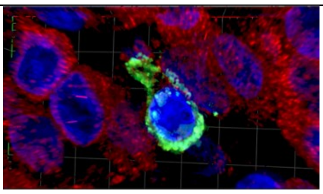 | https://disk.yandex.ru/d/jy8L9Xuv0rtoOQ |
| S41 | Close colocalization of the mast cell nucleus with the nucleus of an atypical melanoma cell | 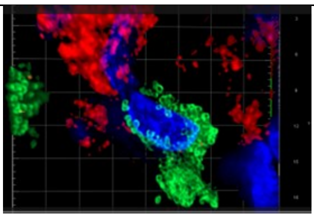 | https://disk.yandex.ru/i/iZHkln1mQ_sKcw |
| S42 | Tryptase-positive cytoplasmic fragments in close proximity to a group of Melan-A-positive tumor cells | 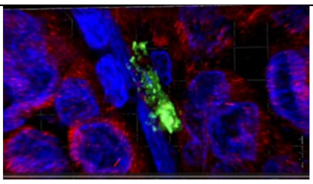 | https://disk.yandex.ru/d/f607hFplIEw6pw |

| | | | |
|---|---|---|---|
| S43 | Loss of mast cell nucleus | 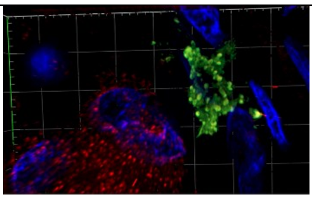 | https://disk.yandex.ru/d/ZYjciOvw6IA4LA |
| S44 | Fragments of tryptase-positive cytoplasmic structures targeting atypical melanoma cells | 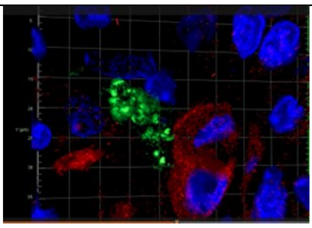 | https://disk.yandex.ru/d/K3gaLZmuCSiRgw |
| S45 | Two large cytoplasmic scaffolds, maintaining autonomous secretion of tryptase to atypical cells | 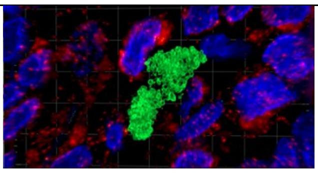 | https://disk.yandex.ru/d/hSAcGqOLfkUdYA |
| S46 | Targeted interaction variants of tryptase transported in granules against atypical melanoma cells | 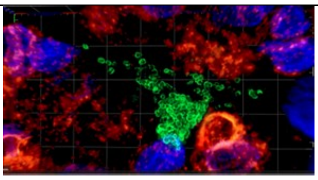 | https://disk.yandex.ru/i/tPUhlBE4jsccyA |
| S47 | | 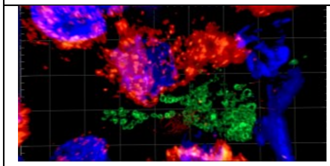 | https://disk.yandex.ru/i/CXQZGt0nOMPi4w |
